# Supplementary material for: Invasive Aspergillosis among Lung Transplant Recipients during Time Periods with Universal and Targeted Antifungal Prophylaxis—A Nationwide Cohort Study
Source: J Fungi (Basel). 2023 Nov 4;9(11):1079. doi: 10.3390/jof9111079 (PMC10672607; doi:10.3390/jof9111079)

Supplementary material

Table S1. Detailed overview of ISHLT classification and specification of causative pathogens identified

	**Universal prophylaxis period, 2010-2016 (IA N=27)**	**Targeted prophylaxis period, 2016-2019 (IA N=15)**
ISHLT classification, n (%)		
**Proven pneumonia**	4 (15)	3 (20)
**Proven tracheobronchitis**	4 (15)	0
**Proven anastomosis infection**	3 (11)	4 (27)
**Probable pneumonia**	11 (41)	4 (27)
**Probable tracheobronchitis**	5 (19)	1 (7)
**Probable anastomosis infection**	0	3 (20)
Pathogens specified, n (%)		
Aspergillus fumigatus	18 (67)	11 (73)
Aspergillus niger	2 (7)	0
**Unspecified mold**	1 (4)	2 (13)
***Aspergillus* species**	3 (11)	0
Aspergillus nidulans	1 (4)	0
Aspergillus ungius	1 (4)	0
**No pathogen specified**	3 (11)	2 (13)

No pathogen was specified in some patients with a proven diagnosis where the diagnosis relied on histopathological findings exclusively.

Abbreviations: Universal antifungal prophylaxis period = Voriconazole three months following transplantation for all patients; Targeted antifungal prophylaxis = Posaconazole and inhaled liposomal amphotericin B three months following transplantation for high IA risk patients; IA = invasive aspergillosis; ISHLT = International Society for Heart and Lung Transplantation.

Table S2. Adjusted hazard ratios (aHR) of first *Aspergillus* event (colonization or invasive aspergillosis) in lung transplant recipients

	**aHR (95% CI)**
Model 1	
**Targeted prophylaxis time period, 2016-2019**	Ref.
**Universal prophylaxis time period, 2010-2016**	0.92 (0.58-1.48)
Model 2	
**Person time without antifungal prophylaxis**	Ref.
**Person time on antifungal prophylaxis**	0.50 (0.24-1.00)

Multivariable Cox models on invasive aspergillosis (IA), Model 1 and 2 included the following variables: Sex, age >50 years, Aspergillus pre-transplantation, high risk of IA, single lung transplantation and prophylaxis guideline period. Model 2 also included person time on prophylaxis as a time-updated variable.

Figure S1. Cumulative hazards of first *Aspergillus* event (colonization or invasive aspergillosis) the first year after lung transplantation in time periods with universal versus targeted prophylaxis

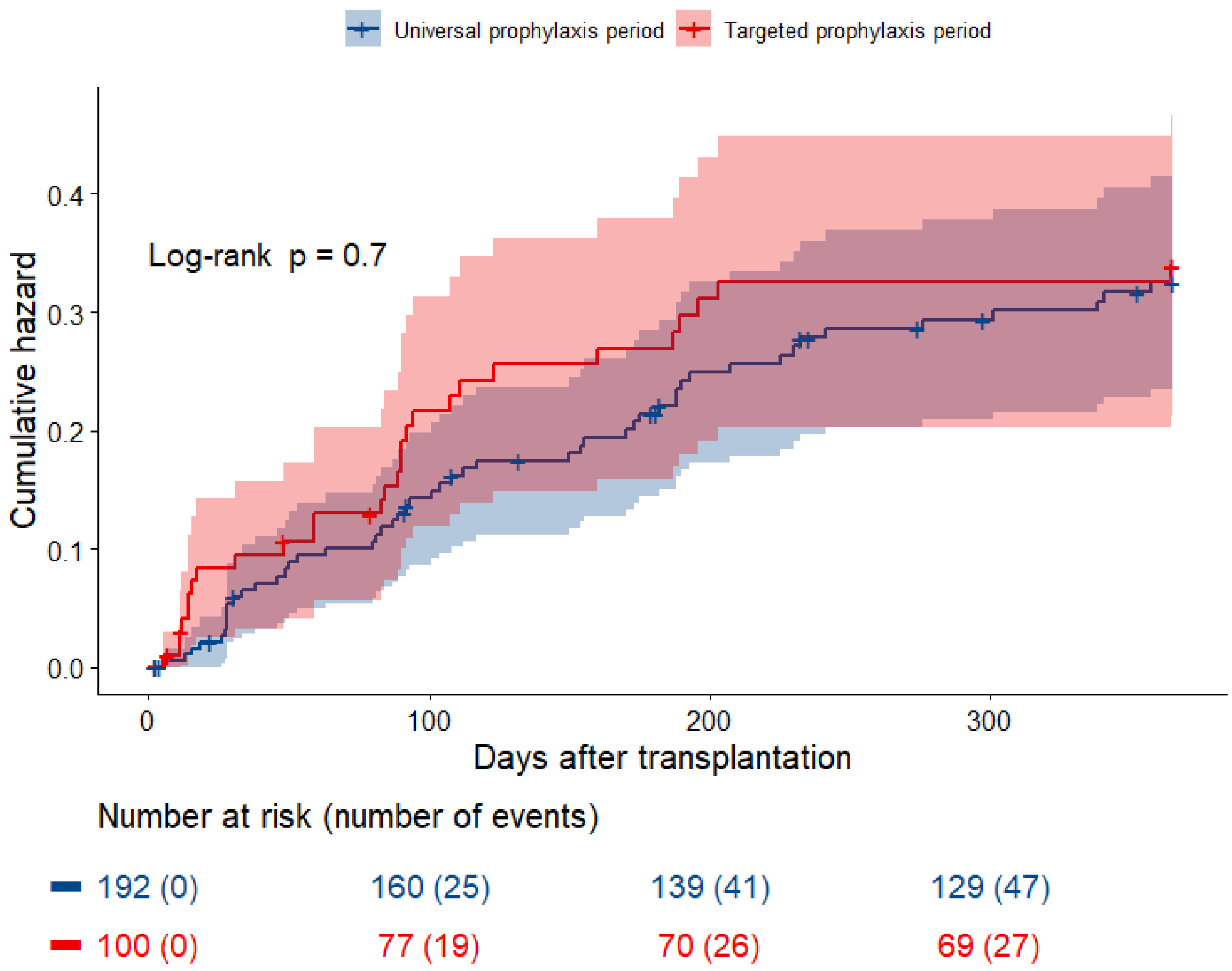

Supplement: Supplementary file 1 [file jof-09-01079-s001.zip › jof-2660980-supplementary.pdf]
